# The impact of depressive symptoms on 5-year all-cause mortality in patients with parkinson’s disease treated with and without deep brain stimulation

**DOI:** 10.1007/s00702-026-03102-6

**Published:** 2026-01-16

**Authors:** Sarah Marschoun, Marco Treven, Christoph Brücke, Johann Lehrner

**Affiliations:** Department of Neurology, Medical University of Vienna, Vienna, Austria

**Keywords:** Parkinson’s disease, Deep brain stimulation, Depression, 5-year all-cause mortality

## Abstract

This retrospective single-center study evaluated the impact of depressive symptoms on survival in patients with Parkinson’s disease (PD) treated with deep brain stimulation (DBS) or best medical treatment (BMT). A total of 421 patients (337 BMT, 84 DBS) from the Medical University of Vienna were included, with data collected between 1999 and 2024. Depressive symptoms were assessed using the Geriatric Depression Scale (GDS), and cognitive performance was evaluated using subtests of the Neuropsychological Test Battery Vienna (NTBV). A Cox proportional hazards model was applied to examine the predictive value of depressive symptoms and additional covariates, including age, gender, education, and cognitive function. Increasing age and male sex were significant risk factors for increased mortality. While depressive symptoms showed an initial association with survival, their predictive strength diminished when cognitive and demographic factors were included. Cognitive performance, particularly attention and executive functioning, assessed by NTBV-15, showed significant protective effects. Five-year survival was 73.4% in the BMT group and 88.2% in the DBS group (*p* < .05), though therapy type itself was not a significant predictor in the regression model. These findings suggest that depressive symptoms may contribute to mortality in PD patients but appear to be outweighed by cognitive and demographic variables. Cognitive assessment may offer additional prognostic value in long-term outcome prediction.

## Introduction

Parkinson’s disease is a progressive neurodegenerative disorder that affects both motor and non-motor domains, and its global burden has more than doubled in the past two decades due to an aging population and extended life expectancy (Reich and Savitt 2019; Tysnes and Storstein 2017). While the cardinal motor symptoms such as bradykinesia, rigidity, and tremor are central to diagnosis and management, non-motor symptoms—especially depression—are increasingly recognized as critical factors that influence disease progression and patient outcomes (Reich and Savitt 2019; Samanta et al. 2023). Depression is one of the most prevalent non-motor symptoms in PD, affecting up to 50% of patients, and contributes significantly to reduced quality of life, faster cognitive and motor decline, and potentially increased mortality (Wu et al. 2017; Weintraub et al. 2003).

Despite the high prevalence of depression in PD, its role in predicting mortality remains controversial. Several studies have suggested associations between depressive symptoms and increased mortality rates in PD patients, yet findings are inconsistent and often confounded by overlapping symptomatology and methodological variability (Hughes et al. 2004; Burn 2002). A recent large-scale cohort study confirmed that PD patients have increased all-cause mortality compared to controls, with particularly strong associations for suicide, dementia, and pneumonia, and found the association to be more pronounced in patients without comorbidities, depression, or dementia (Chiou et al. 2025). Depression may present atypically in PD, often characterized by apathy, irritability, and sleep disturbances rather than classical depressive features such as guilt or suicidal ideation, complicating both diagnosis and treatment (Yamamoto 2001; Farabaugh et al. 2011).

DBS has become a well-established neurosurgical treatment for advanced PD, particularly effective in reducing motor fluctuations and medication-associated complications (Abusrair et al. 2022; Church 2021). While the primary indication for DBS remains motor symptom control, its impact on non-motor symptoms such as mood and cognition is less clear and remains the subject of ongoing investigation (Cartmill et al. 2021). Some studies suggest that DBS may alleviate depressive symptoms in select patients by modulating limbic circuits, whereas others report mood destabilization and even increased depressive symptoms post-surgery, particularly when preoperative psychiatric comorbidities exist (Tysnes and Storstein 2017; Strutt et al. 2012).

Earlier studies also reported possible negative outcome after DBS for semantic and phonematic verbal fluency and a mild trend for a deterioration of verbal memory (Foki et al. 2017, Foki et al. 2018, Harati et al. 2013).

Additionally, the question of whether DBS influences long-term mortality in PD patients remains unanswered. Although improvements in motor function and quality of life are well-documented, evidence regarding DBS’s effect on survival is inconclusive, with studies yielding mixed results (Weaver et al. 2017; Lilleeng et al. 2014). Given the multifactorial nature of mortality in PD - where age, sex, cognitive status, and comorbidities all play essential roles - clarifying the independent contribution of depressive symptoms and DBS treatment to survival outcomes is of high clinical relevance (Forsaa et al. 2010; Kuusimäki et al. 2022; Macleod et al. 2014).

The present study aims to explore whether depressive symptoms, measured using the Geriatric Depression Scale (GDS-15), are predictive of 5-year all-cause mortality in PD patients treated with and without DBS. It further seeks to evaluate whether DBS treatment modifies the relationship between depression and mortality, thereby contributing valuable insights into both prognostic assessment and therapeutic decision-making in the management of Parkinson’s disease.

## Materials and methods

This study represents an university-based, single-center, retrospective data analysis. Data protocols were derived from patients of the Neurological Department of the Medical University of Vienna from the period February 1999 to May 2024. Patient-related data were extracted from the Research Documentation and Analysis (RDA) system and the Allgemeines Krankenhaus Information Management (AKIM) system of the Medical University of Vienna. Information on deceased individuals was available via Statistik Austria and AKIM up to December 2023. Censored cases were included based on the last documented contact until December 2024, enabling calculation of individual survival times.

The study employed a cross-sectional design, categorizing patients into two cohorts: one receiving only best medical treatment and the other receiving both best medical treatment and Deep Brain Stimulation.

### Patient collective

The sample size within the study collective comprised *N* = 421 patients, aged 30 to 86 years. Of these, 337 (80%) received BMT and 84 (20%) underwent additional DBS. Patients had to meet specific inclusion and exclusion criteria; a confirmed diagnosis of Parkinson’s disease according to the MDS clinical diagnostic criteria for Parkinson’s Disease (Postuma et al. 2015). For inclusion in the DBS subgroup, additional criteria were required: a disease duration of at least five years, a positive response to L-DOPA or apomorphine, the presence of motor complications unresponsive to oral pharmacotherapy, or a tremor resistant to standard treatments. Exclusion criteria encompassed the presence of atypical or secondary parkinsonism, severe cognitive impairment, and structural brain lesions or medical comorbidities contraindicating surgical intervention.

### Instruments

All participants completed clinical and neuropsychological assessments, including medical and PD-specific history, neurological examination, blood tests, and evaluation of motor function using the UPDRS-III or the MDS.UPDRS III (introduced in 2008) and Hoehn & Yahr scale. Cognitive function was assessed with the Mini-Mental State Examination (MMSE) and the Neuropsychological Test Battery Vienna (NTBV), assessing attention, executive function, language, and memory. Sociodemographic and biometric data were considered, and depressive symptoms were evaluated using the GDS-15.

Global cognition was screened with the MMSE, a brief inventory evaluating orientation, memory, attention, language, and executive function, with a maximum score of 30 points (Folstein et al. 1975). Visuoconstructive abilities were examined using the Vienna Visuo-Constructional Test (VVT 3.0 Screening), which involves reproducing geometric figures (Lehrner 2025b). Depressive symptoms were assessed using the 15-item version of the GDS, with scores ≥ 5 indicating the presence of depressive symptoms (Mgbeojedo et al. 2022). For a more detailed cognitive profile, the NTBV-15 was employed, assessing domains such as attention, executive function, language, and memory through subtests (Lehrner 2025a). Motor function was evaluated with Part III of the Movement Disorder Society–Unified Parkinson’s Disease Rating Scale (MDS-UPDRS-III), focusing on bradykinesia, rigidity, tremor, and postural stability, (Goetz et al. 2008) and with the Hoehn & Yahr staging system, which categorizes motor severity across five stages. All assessments were conducted preoperatively in the DBS cohort (Hoehn and Yahr 1967).

### Statistical analysis

Descriptive and inferential statistical analyses were conducted using IBM SPSS^®^ Statistics 29.0.2. Hypothesis testing was performed with a significance level of α = 0.05 in accordance with the Type I error rate; p-values ≤ 0.05 were considered statistically significant, and all tests were conducted two-tailed. Effect size measures (*r* and *d*) according to Cohen’s classification were utilized to interpret the practical relevance of results (Cohen 1988).

Descriptive statistics included arithmetic means (*M*), standard deviations (*SD*), minima, maxima, medians (*Mdn*), and interquartile ranges (IQR, Q1–Q3), depending on data distribution. Using boxplots and scatterplots (with regression functions), metric variables were illustrated. Categorical variables were presented using frequencies (n), percentages (%). Baseline characteristics were compared between treatment groups (DBS vs. BMT), including sociodemographic, clinical, and cognitive parameters.

Inferential analyses utilized non-parametric Mann-Whitney U-tests for ordinal or non-normally distributed metric variables (Weiss^ 2019^) and Welch’s t-tests for normally distributed data with unequal variances (considering Levene’s statistics) (Kubinger 2009).

Chi-square testing examined associations between categorical variables; Fisher’s exact test was applied if more than 20% of expected frequencies in contingency tables were below 5 (Bühl 2012). Pearson’s correlation coefficients were used to assess linear relationships between two metric variables (Weiß 2019). To reduce dimensionality and examine the factorial structure of the NTBV-15 short version, a principal component analysis (PCA) with orthogonal Varimax rotation (Kaiser’s criterion) was carried out (Backhaus 2021). The Kaiser-Meyer-Olkin (KMO) coefficient assessed sampling adequacy (KMO ≥ 0.50 acceptable, ≥ 0.80 substantial) (Hatzinger and Nagel 2009). Components with eigenvalues ≥ 1 were retained; communalities and scree plot inspection supported interpretability. Factor scores were *z*-standardized (µ = 0, σ = 1) and uncorrelated (*r* = 0), enabling use in subsequent analyses as weighted indices for neurocognitive domains (Backhaus 2021).

Survival likelihood analyses were conducted using the Kaplan-Meier (KM) method, considering censored cases and varying follow-up durations (Ziegler et al. 2007c). Group comparisons were performed via log-rank tests (Ziegler et al. 2007b). To examine the influence of covariates on survival time, a Cox proportional hazards regression model was applied, reporting hazard ratios (HR) and 95% confidence intervals (Ziegler et al. 2007a).

### Analysis results

Our sample of *N* = 421 patients also demonstrated that the disproportionate ratio of male patients (62.5% 95%-CI [56.6%; 68.3%]) could be confirmed in the context of PD. The median age of the entire study population was 68.5 (min 30.6 - max 90.5) years.

There were significant differences with moderate effects with regard to age at onset (in years), DBS 48 vs. BMT 63 and duration (in years) DBS 9.8 vs. BMT 5.9. Furthermore, BMT patients revealed a significant higher UPDRS III score, *p* <.001 with a small difference (effect size *r* =.24) compared to the DBS group indicating a mobility restriction.

A comparison of the two PD treatment groups revealed a significant difference in the distribution of Hoehn & Yahr stages, showing the progression of the disease *p* <.001. The results suggest that BMT patients were more frequently classified in higher Hoehn & Yahr stages.

**Fig. 1:**
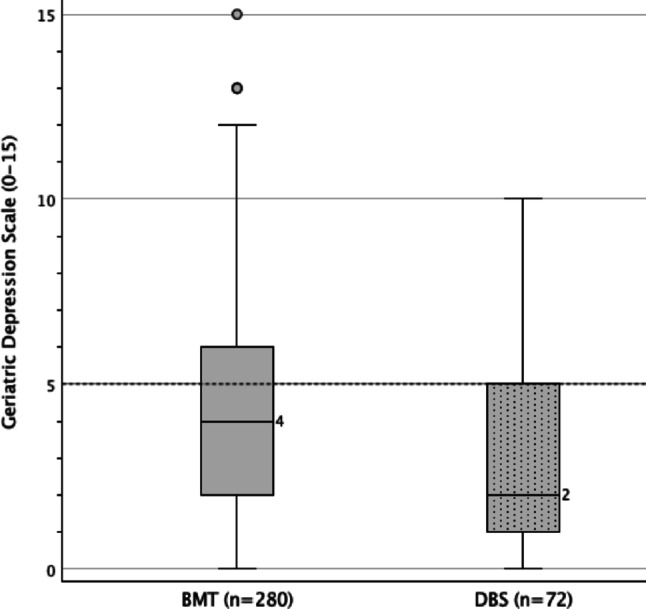
Distribution of GDS-15 scoring considering the two PD treatment subgroups, taking valid protocols (n) into account

MMSE showed elevated cognitive performance in the DBS group, with a small effect size (*r* = + 0.19). In contrast, no significant difference was found in visuo-constructive abilities assessed by the VVT 3.0 Screening (*r* = –.03). The DBS group also showed significantly better performance across all 15 NTBV subtests. After dimensional reduction using principal component analysis (PCA), superior results remained evident in the four extracted cognitive domains—attention, verbal memory, executive functioning, and naming & verbal fluency—with effect sizes ranging from d = + 0.22 to + 0.48. Depressive symptoms, measured by the GDS-15, were significantly more pronounced in the BMT group compared to the DBS group, regarding a small effect (*r* = –.12), as shown in Fig. 1. Overall, 36.9% of the total study collective exhibited clinically relevant depressive symptoms, 95% CI [31.9%; 42.0%], taking the cut off score ≥ 5 into account.

**Fig. 2:**
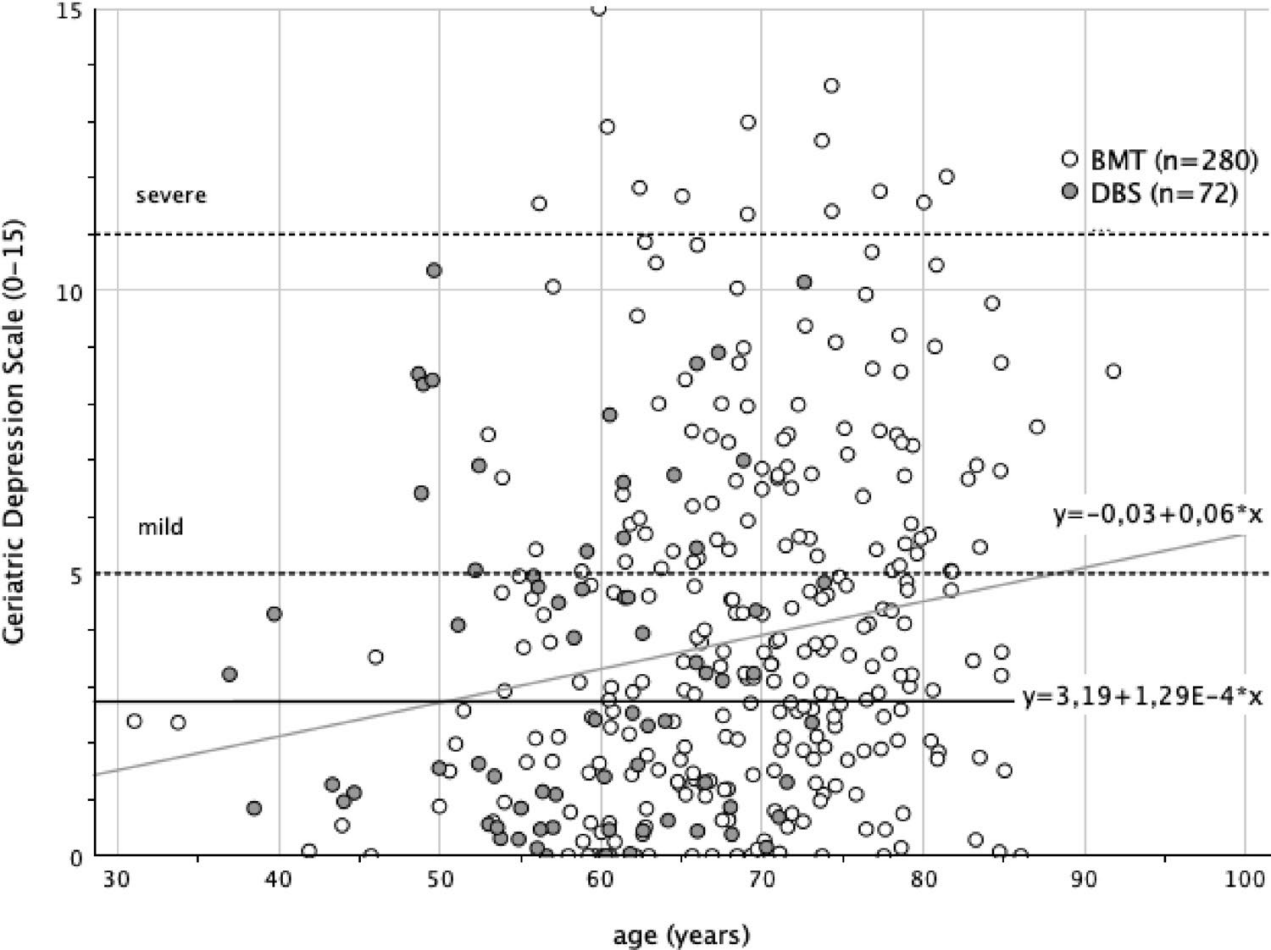
Bivariate scatterplot with linear regression functions and equations considering age at testing and GDS scoring regarding the two PD subgroups

A positive, significant relation between depression and age was observed in BMT patients, *r* =.17 (*p* =.005, two tailed, *n* = 280), whereas there was no such association in DBS patients, *r* =.00 (*p* =.998, two tailed, *n* = 72). It can be assumed that depression increases with age only within BMT patients. For this group, a linear regression equation of Ŷ = −0.03 + (0.06 * xi) was found, so that an increase of 0.6 GDS points per decade can be assumed as shown in Fig. 2.

The survival likelihood, considering the follow up time using the Kaplan-Meier approach, showed the following results comparing the two PD subgroups in Table 1.

**Table 1:** Survival likelihood of PD subgroups up to 5 years using the KM approach

PD subgroup	6 months	1 year	2 years	3 years	5 years
BMT *n* = 337	98.7%	97.1%	92.8%	84,9%	73.4%
DBS *n* = 84	100%	98.8%	96.3%	96.3%	88.2%
Total *N* = 421	99.0%	97.5%	93.6%	87.2%	76.3%

**Fig. 3:**
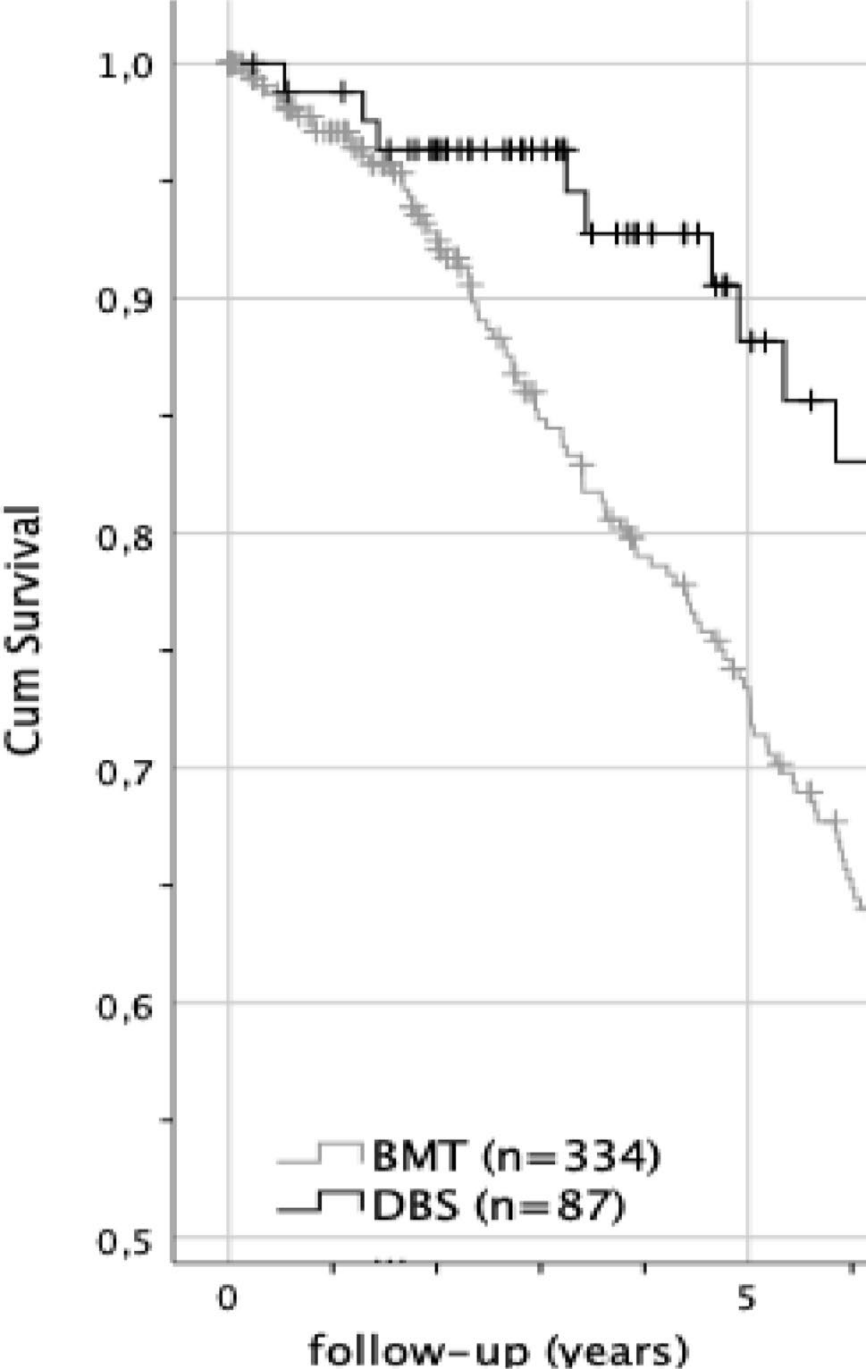
KM survival functions of BMT and DBS PD patients

The log-rank test revealed a significant result, χ^2^ (1) = 18.546, *p* <.001. It can be assumed that, considering the follow-up period, DBS patients have a 14.8% higher cumulated 5-year survival likelihood compared to BMT (88,2% vs. 73,4%), as illustrated in Fig. 3.

The main analysis was conducted using a Cox proportional hazards regression model to examine the predictive value of relevant variables for mortality (0 = censored, 1 = deceased), taking into account the follow-up time. A hierarchical blockwise entry of predictors was applied across five blocks: Block 1 included depressive symptomatology (GDS, *continuous*); Block 2 added sex (0 = male, 1 = female, *binary*) and age (in years, *continuous*); Block 3 incorporated formal education (years of schooling, *continuous*); Block 4 comprised the z-standardized factor scores derived from the NTBV-15; and Block 5 introduced the PD treatment group (0 = BMT, 1 = DBS) as a dichotomous predictor, as shown in Table 2.

**Table 2:** Key values of predictors and covariates for the binary criterion deceased (1) vs. censored (0) considering follow up time (*n* = 336 cases with complete documented protocols)

Block	Predictor	B	SE	Wald χ^2^ (df = 1)	*p*-value	HR	95%-CI HR	
							LB	UB
1	GDS	0.081	0.023	12.494	< 0.001^**^	1.084	1.037	1.133
2	GDS	0.044	0.024	3.233	0.072°	1.045	0.996	1.096
	sex	− 0.567	0.159	12.724	< 0.001^**^	0.567	0.415	0.775
	age (years)	0.091	0.011	73.104	< 0.001^**^	1.095	1.073	1.118
3	GDS	0.043	0.025	3.129	0.077°	1.044	0.995	1.096
	sex	− 0.585	0.160	13.392	< 0.001^**^	0.557	0.407	0.762
	age (years)	0.090	0.011	71.639	< 0.001^**^	1.095	1.072	1.118
	Years of formal eduation (YFE)	− 0.021	0.021	1.034	0.309	0.979	0.941	1.020
4	GDS	0.013	0.026	0.244	0.621	1.013	0.963	1.066
	sex	− 0.715	0.177	16.301	< 0.001^**^	0.489	0.346	0.692
	age (years)	0.071	0.012	34.181	< 0.001^**^	1.073	1.048	1.099
	Years of formal eduation (YFE)	0.008	0.022	0.134	0.714	1.008	0.966	1.051
	F1 Attention	− 0.471	0.084	31.532	< 0.001^**^	0.624	0.530	0.736
	F2 Verbal memory	− 0.042	0.093	0.205	0.651	0.959	0.799	1.150
	F3 Executive function	− 0.171	0.079	4.681	0.030^*^	0.843	0.722	0.984
	F4 Naming & verbal fluency	− 0.103	0.085	1.475	0.225	0.902	0.763	1.065
	GDS	0.013	0.026	0.248	0.619	1.013	0.963	1.066
	sex	− 0.703	0.178	15.668	< 0.001^**^	0.495	0.350	0.701
	age (years)	0.068	0.013	28.785	< 0.001^**^	1.070	1.044	1.097
	Years of formal eduation (YFE)	0.009	0.022	0.166	0.683	1.009	0.967	1.052
5	F1 Attention	− 0.477	0.084	32.230	< 0.001^**^	0.621	0.527	0.732
	F2 Verbal memory	− 0.044	0.093	0.222	0.638	0.957	0.797	1.149
	F3 Executive function	− 0.172	0.079	4.780	0.029^*^	0.842	0.722	0.982
	F4 Naming & verbal fluency	− 0.105	0.085	1.517	0.218	0.901	0.763	1.064
	PD treatment (BMT vs. DBS)	− 0.215	0.269	0.635	0.426	0.807	0.476	1.368

^**^p ≤.01, ^*^p ≤.05, °*p* ≤.10 (tendency)

The results indicate that, in the first model block, depressive symptoms (GDS) show a small but statistically significant association with mortality, HR = 1.084, *p* <.001. However, upon inclusion of the biometric covariates age and gender in the second block, the predictive weight of depression diminishes. Age remains a consistent and significant predictor throughout all model blocks, with HR = 1.070, *p* <.001, as does male gender (inverted coding), HR = 2.020, *p* <.001. Years of formal eduation (YFE), did not contribute a significant effect on mortality. In contrast, two cognitive dimensions derived from the NTBV-15, Factor 1 (Attention), HR = 0.621, *p* <.001, and Factor 3 (Executive Functioning), HR = 0.843, *p* =.029, demonstrated a statistically significant protective effect considering mortality. In the final model block, the inclusion of the treatment modality (0 = BMT, 1 = DBS) did not reveal a significant contribution to mortality prediction, HR = 0.807, *p* =.426.

## Discussion

This study investigated the relationship between depressive symptoms and five-year mortality in patients with PD, comparing those treated with BMT to those who received additional DBS. Depression is one of the most frequent non-motor symptoms in PD, affecting quality of life and possibly increasing mortality risk (Hughes et al. 2004; Weintraub et al. 2003).

Initial analyses revealed that depressive symptoms, assessed using the GDS, were more prevalent in the BMT group (38.6%) compared to the DBS group (30.6%), with a weak effect size (*r* = –.12). Severe depression (GDS ≥ 11) was only observed in the BMT group (5.7%). These findings may be influenced by selection bias, as DBS candidates are generally younger and in better psychological condition (Dallapiazza et al. 2018). Moreover, patients may underreport depressive symptoms in order to not jeopardize DBS eligibility.

The total depression prevalence (36.9%) in this sample was slightly below the typical range of 40–50% reported in the literature (Wu et al. 2017; Weintraub et al. 2003). This could be partly explained by the younger mean age of the DBS group and by differences in depression assessment tools.

Neuropsychological testing revealed better cognitive performance in the DBS group, particularly in attention (r = + 0.48), verbal memory (r = + 0.45), executive functioning (r = + 0.22), and verbal fluency (r = + 0.47). These differences can be attributed to the preselection of DBS candidates, who are generally cognitively intact and thus suitable for surgery (Du et al. 2022).

In terms of survival, the five-year survival rate was higher in the DBS group (88.2%) compared to the BMT group (73.4%), as shown by Kaplan-Meier analysis. However, in the Cox regression model, the type of treatment was not a significant predictor of mortality. Instead, increasing age and male gender emerged as the most relevant risk factors, consistent with earlier findings by Forsaa et al. (Forsaa et al. 2010). These demographic variables appear to outweigh the contribution of depressive symptoms when predicting mortality.

Interestingly, the impact of depression on mortality also appears to be mediated by age. While age correlated positively with depression in the BMT group (r = + 0.17), no such relationship was observed in the DBS group (*r* = 0), suggesting age-related depressive burden is more pronounced among non-DBS patients. Cognitive performance in BMT patients showed a weak negative correlation with depression (*r* = –.06 to –0.32), whereas no consistent pattern emerged in the DBS group.

Further, higher cognitive test scores in the domains attention and executive function were identified as protective factors in the regression model. This highlights the prognostic relevance of non-motor features in PD, particularly cognitive resources that support daily function and treatment adherence. These findings align with previous studies that emphasized the protective role of attention in cognitively impaired populations (Đapić et al. 2022; Dreier et al. 2023; Futschek et al. 2023), while executive functioning appears especially relevant in PD, given the complexity of medication and disease management.

While earlier studies reported possible negative outcome after DBS for semantic and phonematic verbal fluency and a mild trend for a deterioration of verbal memory (Foki et al. 2017, Foki et al. 2018, Harati et al. 2013) the present study did not find an effect of verbal fluency and verbal memory on 5-year all-cause mortality.

Although DBS has demonstrated improvements in motor function and quality of life, its effect on mortality remains debated. Weaver et al. reported a potential survival advantage (Weaver et al. 2017), whereas Lilleeng et al. found no significant difference (Lilleeng et al. 2014). Our results support the latter and suggest that while DBS improves quality of life, it may not significantly prolong survival.

The mean age at death in our cohort (78.6 years) closely mirrors that of the general male population in Vienna (78.6 years), but is 4.1 years lower among female PD patients compared to the general female population (83.3 years) (Austria 2023). This gender difference in disease burden and mortality underscores the need for gender-sensitive research and care strategies in PD.

In conclusion, while depressive symptoms are associated with higher mortality in PD, they do not independently predict survival when controlling for age, gender, and cognitive functioning. Cognitive variables and demographic factors were more powerful predictors of five-year mortality than treatment type or depression alone. These findings highlight the necessity of an integrated clinical approach that includes psychiatric and cognitive assessment in addition to motor symptoms, to better inform prognosis and individualized care planning in PD.

### Limitations

Several limitations have to be mentioned. Considering missing data, some analyses were restricted to cases with complete documentation; for instance, 85 cases (20.2%) were excluded from the Cox regression (*n* = 336). Additionally, neuropsychological testing was conducted prior to DBS surgery, meaning subgroup differences cannot be interpreted as DBS effects but rather reflect baseline disparities, limiting the findings to a descriptive and exploratory level. The risk of selection bias is notable, given the non-randomized group allocation and differing disease severity. To improve group comparability for future retrospective studies, matching procedures such as propensity score methods are recommended to control for confounding variables.

### Preview

Future studies should aim to replicate or critically assess these findings, ideally using matched samples to control for confounders. Longitudinal designs are recommended to further examine the absence of an age-depression correlation in DBS patients and to explore depressive symptoms as potential mediators of survival through appropriate regression models.

## Conclusion

A positive association between age and depression was found only in the BMT treatment group underlining age as a confounder. Kaplan-Meier analysis indicated lower five-year survival in BMT (73.4%) compared to DBS (88.2%). Cox regression identified male gender, age, and lower cognitive performance as significant mortality predictors, while depression lost its weight when these were considered. Treatment modality showed no independent effect on mortality.

## Data Availability

The data can be obtained within reasonable request.
